# Breaking the intergenerational cycle of extinction of experience: Actions needed and pathways forward

**DOI:** 10.1017/ext.2026.10015

**Published:** 2026-05-26

**Authors:** Masashi Soga, Kevin J. Gaston

**Affiliations:** 1 Graduate School of Agricultural and Life Sciences, The University of Tokyo, Japan; 2 University of Exeter Environment and Sustainability Institute, UK

**Keywords:** behaviour change, connection to nature, extinction of experience, global change, personalised ecologies

## Abstract

To halt and reverse the ongoing biodiversity crisis, substantial changes in human behaviour will be required. One important challenge in achieving such changes is the ‘extinction of experience’ – the progressive loss of direct human interactions with nature (hereafter ‘personalised ecologies’). Diminished personalised ecologies can erode emotional ties to nature, weaken pro-conservation attitudes and reduce engagement in the diverse actions needed to support biodiversity. Although the extent of this decline has increasingly been documented, far less attention has been paid to the processes through which it unfolds over time, particularly across generations. In this Perspective, we examine how the extinction of experience may become entrenched within societies. We propose a conceptual framework, drawing on ideas from behavioural genetics, to explore how personalised ecologies could be transmitted across generations through genetic, environmental and interactive pathways. We then use this framework to consider why, once initiated, the extinction of experience may persist and intensify over time. Finally, we outline potential strategies to help disrupt this cycle of disconnection and to foster more positive, self-reinforcing trajectories of human–nature interactions. Achieving this will likely require coordinated efforts across sectors, including conservation organisations, education systems, urban planning and public health initiatives.

## Impact statement

Many human societies are losing direct, everyday interactions with nature – so-called ‘personalised ecologies’. This decline, often described as the ‘extinction of experience’, can have wide-ranging consequences for both biodiversity conservation and human health. Whilst evidence for this decline is accumulating, a critical question remains: why do people’s experiences of nature continue to decline across societies? One possibility is that this decline is not only driven by immediate environmental change but also reinforced across generations. As environments become increasingly degraded, individuals with limited personalised ecologies may be more likely to pass on similarly reduced experiences to subsequent generations, creating a self-reinforcing dynamic. To explore this possibility, we use insights from behavioural genetics to examine how personalised ecologies may be transmitted across generations, particularly through family- and household-level processes. From this viewpoint, the intergenerational persistence of declining nature experience can arise through two key pathways: (1) the inheritance of already impoverished personalised ecologies, and (2) the failure to transmit rich personalised ecologies when environmental changes constrain opportunities, motivation and capability to engage with nature. Over time, such reinforcing dynamics may deepen disconnection from nature and make reversal increasingly challenging. Importantly, however, this framework also points to the potential for the opposite dynamic: when opportunities, motivation and capability to engage with nature are supported, rich personalised ecologies may be more likely to persist across generations. Achieving this will likely require coordinated efforts across multiple sectors, including environmental policy, urban planning, education and public health. Transforming current trajectories into more positive, self-reinforcing cycles of human–nature interactions will likely be important for the future of both biodiversity and human well-being.

## Introduction

The world is now thought to have entered into a sixth mass extinction, driven by a wide range of human activities (Ceballos et al., 2015; Cowie et al., 2022). Current estimates suggest that around one million species are at risk of extinction worldwide, emphasising the urgent need for biodiversity conservation (IPBES, 2019). Beyond species loss alone, these ecological changes have profound consequences for human societies, affecting ecosystem services that underpin human health and well-being (Cardinale et al., 2012; Sandifer et al., 2015).

Alongside the biodiversity crisis, and partly as a consequence of it, another form of ‘extinction’ is also occurring – the ‘extinction of experience’ (Pyle, 1993; Miller, 2005; Soga and Gaston, 2016; Gaston and Soga, 2020). This phenomenon refers to the progressive loss of direct, sensory interactions that people have with nature (hereafter ‘personalised ecologies’; Gaston et al., 2018; Gaston, 2024). A growing body of evidence indicates that people, particularly in more urbanised and high-income countries, have been experiencing steady declines in everyday contact with nature over the past few decades (e.g., Pergams and Zaradic, 2008; Imai et al., 2018; Soga et al., 2018; Soga and Gaston, 2023a). Like biodiversity loss, the extinction of experience has negative consequences for both humans and nature: reduced personalised ecologies can undermine health and well-being (see reviews by Keniger et al., 2013; Hartig et al., 2014; Marselle et al., 2021) and weaken support for pro-nature policies and conservation initiatives (Rosa et al., 2018; Alcock et al., 2020; Martin et al., 2020; Soga and Gaston, 2023b; Soga and Gaston, 2024a). Consequently, the extinction of experience is not only considered a public health concern but also a key barrier to global biodiversity conservation (Soga and Gaston, 2016; Gaston and Soga, 2020).

Whilst the extent of temporal decline in personalised ecologies has been documented (e.g., Pergams and Zaradic, 2008; Soga and Gaston, 2016; Imai et al., 2018; Soga et al., 2018; Soga and Gaston, 2023b), far less attention has been given to the processes through which the extinction of experience, and particularly the loss of positive or neutral nature experiences, unfolds over longer timescales, especially across generations. One explanation is that each generation is exposed to a progressively more depauperate biota than the previous one (noting that biodiversity change follows complex and spatially variable trajectories; Blowes et al., 2019). However, beyond such direct environmental change, the extinction of experience may also be transmitted across generations, potentially reinforcing or accelerating these declines. A reduction in personalised ecologies in one generation can alter attitudes and behaviours towards nature (Soga and Gaston, 2016), which in turn may constrain the opportunity, motivation and capability for similar experiences in the next (Soga and Gaston, 2022a). Empirical studies support this view, showing that parents’ attitudes and behaviours strongly shape children’s personalised ecologies, both by directly influencing the scope and accessibility of outdoor activities (e.g., where and how far children are allowed to go) and by indirectly shaping their orientation towards nature (e.g., Hammond et al., 2011; McFarland et al., 2014; Hand et al., 2018; Soga et al., 2018; Van Truong et al., 2023; Pengwei et al., 2025). This suggests that the extinction of experience is not merely an individual phenomenon but can become embedded within familial and social structures.

Recognising that the extinction of experience has an intergenerational dimension, maintaining personalised ecologies will be particularly challenging under growing environmental and societal pressures (Miller, 2005; Soga and Gaston, 2016; Ives et al., 2018; Soga and Gaston, 2024b; Aota et al., 2025). Indeed, the loss of personalised ecologies in even a single generation (and a reduction in their onward transmission) can have lasting consequences, allowing disconnection from nature to persist and deepen in those generations that follow. However, despite their likely importance and widespread relevance, the mechanisms through which personalised ecologies are transmitted across generations, and the conditions under which these processes reinforce or disrupt long-term trajectories of human–nature interactions, remain poorly understood. This is partly because most research to date has focused on the immediate conditions shaping personalised ecologies within a given generation, such as environmental context or socio-demographic factors, rather than on the intergenerational processes through which these experiences are reproduced, maintained or eroded (e.g., Colléony et al., 2020; Garfinkel et al., 2024; Izquierdo et al., 2025; Kamphuisen et al., 2025). As a result, the ways in which personalised ecologies persist, accumulate or decline over longer timescales have received limited systematic attention. Indeed, existing frameworks of human–nature interactions have rarely incorporated these intergenerational linkages explicitly (Soga and Gaston, 2016; Soga and Gaston, 2022a; Gaston, 2024), leaving an important gap in understanding. Without a mechanistic account of these processes, it is difficult to explain why declines in personalised ecologies persist in some contexts but not in others, to anticipate when such declines may accelerate or be delayed, or to design interventions that can effectively sustain human–nature interactions across generations.

In this Perspective, our aim is to develop a conceptual framework for understanding how the extinction of experience is transmitted across generations, and to identify the mechanisms through which such processes may sustain or intensify disconnection from nature over time. To this end, we first propose a framework for the intergenerational transmission of personalised ecologies. We then use this framework to examine how and why the extinction of experience, once initiated, may persist and intensify through intergenerational pathways. Finally, we discuss the broader implications of these dynamics and highlight potential strategies to break this cycle of disconnection at a time when broad-based public support for conservation is urgently required. Throughout, we use the term personalised ecologies to refer to the full range of an individual’s direct sensory interactions with wild organisms (Gaston et al., 2018; Gaston et al., 2023; Gaston, 2024). However, for the purposes of this article, we focus on positive or neutral experiences of nature, as noted above, because the extinction of experience is primarily characterised by, and of concern due to, the decline of such interactions (Pyle, 1993; Miller, 2005; Soga and Gaston, 2016; Gaston and Soga, 2020). Negative experiences of nature (e.g., being bitten by mosquitoes or attacked by a bear; Kasturiratne et al., 2008; Bombieri et al., 2019; Soga and Gaston, 2022b) are often discussed from a different perspective (i.e., their reduction is often a focus or goal in public health and wildlife management contexts) and are therefore not considered here, although the proposed framework could be applied to them.

## Conceptual framework

To examine how personalised ecologies are transmitted across generations, we adopt a behavioural genetics perspective, a branch of psychology that seeks to disentangle and explain genetic and environmental contributions to human behaviour (Plomin et al., 1977; Scarr and McCartney, 1983). This framework has been widely applied across a range of behavioural and psychological traits (e.g., personality, cognitive ability and mental health), providing a useful (albeit still developing) basis for examining the joint roles of genetic and environmental influences (Hambrick and Tucker-Drob, 2015; Schellenberg, 2015; Ullén et al., 2016; Bulbena-Cabre et al., 2018; Hopwood et al., 2022; Herrera-Luis et al., 2024). Whilst the transmission of individuals’ attitudes and behaviours towards nature can also be understood at broader societal and institutional scales (e.g., through education systems and cultural norms), here we focus on processes operating at the level of individuals and families, where intergenerational transmission is most directly experienced and where genetic and environmental influences are most tightly coupled.

**Figure 1.**
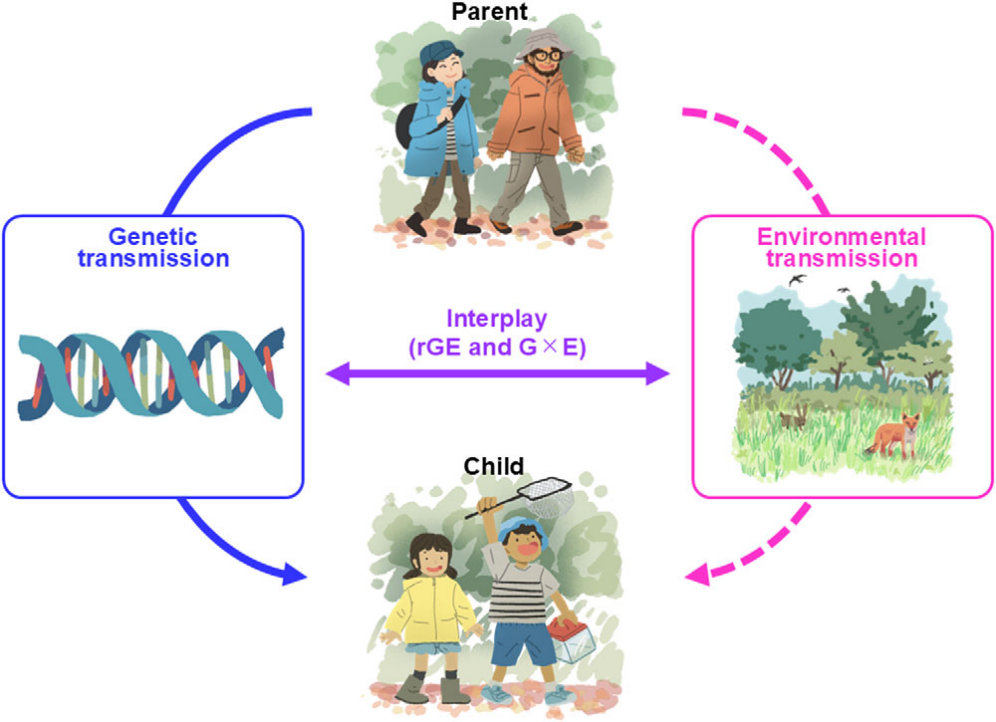
Conceptual framework illustrating how personalised ecologies (the set of an individual’s direct, sensory interactions with nature) can be transmitted across generations. Although such transmission can occur across multiple social and spatial scales, the framework focuses on parent–child relationships, where intergenerational processes are most directly expressed. Transmission can arise through genetic pathways (e.g., heritable traits influencing preferences or sensitivities), environmental pathways (e.g., parental choices of neighbourhoods, leisure activities and social contexts) and their interplay. This interplay may include gene–environment correlation (rGE), whereby genetic predispositions can influence the environments individuals experience, and gene by environment interaction (G × E), whereby the effects of genetic predispositions may depend on environmental conditions. These processes can shape children’s opportunities, motivations and capabilities for engaging with nature. Importantly, environmental influences can extend beyond the family to broader social contexts (e.g., communities, institutions and policies), highlighting that these pathways are not fixed and may be modified through targeted interventions.

From this perspective, personalised ecologies can be viewed as a behavioural phenotype shaped by both genetic and environmental influences, as well as their interplay (Plomin et al., 1977; Scarr and McCartney, 1983; Knopik et al., 2016; Figure 1). We acknowledge that direct empirical evidence supporting the application of this behavioural genetics perspective to the intergenerational transmission of personalised ecologies remains limited, particularly with respect to genetic influences (see below). Accordingly, our aim is to advance a conceptual perspective for exploring intergenerational processes that have received relatively little attention, rather than to synthesise existing evidence into a definitive or fully established, and particularly quantitative, mechanistic understanding.

Below, we discuss the environmental and genetic pathways in more detail. To clarify how these influences translate into patterns of nature experience, we draw on the COM-B framework (Michie et al., 2011), conceptualising these pathways as shaping individuals’ capability, opportunity and motivation to engage with nature, which in turn influence the formation and persistence of personalised ecologies across generations.

### Environmental pathways

Environmental pathways describe how similarities in personalised ecologies between generations arise through shared surroundings. These pathways encompass both physical and social environments (Soga and Gaston, 2022a). Physical factors include the accessibility of green spaces (Neuvonen et al., 2007; Soga et al., 2015; Spotswood et al., 2021; Lee et al., 2023) and the abundance of plants and animals (especially common species) in the local environs of a person (Hand et al., 2017; Cox et al., 2019; Gaston, 2023; Garfinkel et al., 2024), both of which determine the frequency and ease of encounters with the rest of the natural world. Social factors operate across multiple scales. At the family level, parents’ attitudes and behaviours towards nature shape children’s early experiences. For example, parents who are uncomfortable in natural settings or place low value on interacting with nature may be less likely to encourage outdoor play or engagement with wildlife. Such family environments can reduce children’s motivation and opportunity to interact with nature, reinforcing similarities in personalised ecologies across generations. At broader scales, social factors include prevailing cultural attitudes and community norms regarding outdoor play and interaction with nature (Yoon and Lee, 2019; Loebach et al., 2021), as well as the extent to which schools emphasise nature-related knowledge and experiences (Yamanoi et al., 2021).

Intergenerational similarity in environmental conditions can arise from two key processes: environmental stability and non-random mobility. Environmental conditions in which people are embedded, both physical (e.g., urban form, access to nature) and social (e.g., local norms, institutional settings), often change gradually over time at multiple scales (e.g., communities, regions, countries), resulting in a degree of temporal continuity across generations (Zhao et al., 2013). In addition, mobility is rarely random and can generate intergenerational similarity through at least two distinct pathways. First, individuals often remain geographically close to where they grew up or to their parents (Kolk, 2017; Spring et al., 2017), maintaining exposure to similar environments across generations. Second, even when individuals relocate over longer distances, moves are frequently structured by socioeconomic factors, preferences and constraints, leading people to select environments that are broadly similar in their physical and social characteristics (e.g., neighbourhood type) (Van Ham et al., 2014; Hedman et al., 2015). Of course, the extent to which these two processes contribute to intergenerational similarity is likely to vary across contexts, depending on broader factors such as urban planning and social policy.

To the extent that environmental conditions are maintained across generations, they can have important implications for understanding inequalities in personalised ecologies. Because environmental conditions and opportunities for nature experience are unevenly distributed, their continuity across generations can lead to the accumulation and amplification of disparities in nature experiences over time. This pattern is consistent with the ‘luxury effect’, whereby access to biodiversity and green space is disproportionately concentrated in more affluent areas (Leong et al., 2018). As a result, individuals in resource-rich environments are more likely to maintain or accumulate richer personalised ecologies across generations, whereas those in resource-poor environments may experience persistent deficits in opportunities for nature interaction. Given that personalised ecologies are associated with a wide range of positive health outcomes (Keniger et al., 2013; Hartig et al., 2014; Marselle et al., 2021), these intergenerational disparities in experience may, in turn, contribute to broader inequalities in human health. Addressing this challenge will likely require urban planning and social policies that promote more equitable access to nature and support engagement across communities and generations.

### Genetic pathways

Personalised ecologies may also be shaped, in part, by genetically influenced traits, although their role remains much less well understood than that of environmental pathways. A range of physical and psychological characteristics that influence a person’s capability and motivation to engage with nature show evidence of heritability and may therefore contribute to intergenerational patterns in nature experience.

From a physical perspective, traits that facilitate interactions with nature, such as sensory acuity (e.g., vision and hearing) and physical fitness, are known to have genetic components (Silventoinen et al., 2008; Lopes et al., 2009; Mutti, 2010; Schutte et al., 2016). These traits can influence both the ability to perceive elements of the natural environment and the capacity to participate in outdoor activities. In addition, there is emerging, although still limited, evidence that psychological characteristics relevant to human–nature interactions may also have a genetic basis. For example, an individual’s inclination to seek contact with nature (often discussed in relation to the concept of biophilia; Wilson, 1986; Kellert and Wilson, 1995) has been argued to reflect, at least in part, evolved predispositions shaped by past selection pressures (Fukano and Soga, 2024), and there is some initial evidence suggesting that variation in such tendencies may be partly influenced by genetic factors (Chang et al., 2022a, 2022b). Conversely, negative responses to nature, such as fear or aversion (often referred to as biophobia), may act to reduce engagement with natural environments (Soga et al., 2023; Soga and Evans, 2024), and there is some evidence that these responses may also have heritable components (e.g., Hettema et al., 2003; Van Houtem et al., 2013).

The empirical evidence for genetic influences on people’s emotions and attitudes towards nature remains limited and is still subject to considerable debate (see Fukano and Soga, 2025; Joye and De Block, 2025). Nevertheless, given the substantial body of research demonstrating genetic contributions to a wide range of human behavioural and psychological traits (Vukasović and Bratko, 2015; Arancibia et al., 2025), it is plausible that affective responses to the natural world (responses that have likely been a fundamental part of human experience throughout our evolutionary history) also have, at least in part, a genetic basis (Fukano and Soga, 2024). Clarifying the extent to which such responses are genetically influenced will be an important direction for future research.

### Gene–environment interplay

Beyond the independent effects of genes and environments, personalised ecologies may also be shaped by their interplay (Figure 1). From a behavioural genetics perspective, two key processes are likely to be involved: gene–environment correlation (rGE), whereby genetic propensities influence the environments that individuals experience, and gene by environment interaction (GxE), whereby the effects of genetic predispositions depend on environmental conditions.

rGE encompasses processes by which the genetic propensities of individuals affect the kinds of environments they experience. It is classified into three types: passive, active and evocative (Plomin et al., 1977; Scarr and McCartney, 1983; Knopik et al., 2016). *Passive* rGE occurs when parents provide both genes and environments to their children. For example, parents who have a strong interest in nature are more likely to spend time outdoors themselves (Chang et al., 2022a) and to enrich their surroundings with biodiversity, such as by wildlife-friendly gardening (Samus et al., 2023; Hoggett et al., 2024), thereby raising their children in settings that are richer in nature. In this case, children inherit both the genetic inclination and the nature-rich environment from their parents, reinforcing their personalised ecologies. *Active* rGE refers to individuals actively selecting or creating environments that match their genetic tendencies. A child with a heritable preference for outdoor activity might seek out natural environments, even if not strongly encouraged by parents. Over time, this self-selection reinforces their personalised ecologies. *Evocative* rGE involves a child’s genetically influenced traits eliciting certain responses from the environment. For instance, a child who exhibits a natural enthusiasm for animals may elicit greater encouragement or support from parents to participate in outdoor or nature-related activities, thus enhancing their personalised ecology through interpersonal feedback.

GxE refers to processes in which the influence of genetic predispositions on personalised ecologies depends on environmental context (Plomin et al., 1977; Knopik et al., 2016). In other words, similar genetic tendencies may lead to different outcomes depending on the environments in which individuals are embedded. For example, a predisposition towards curiosity about nature may translate into frequent nature experiences in environments rich in accessible biodiversity, but may remain largely unrealised in highly urbanised settings with limited opportunities for interaction. Conversely, supportive environments, such as schools that promote outdoor learning or communities with strong pro-nature norms, may amplify even modest genetic inclinations towards nature engagement.

Personalised ecologies are likely shaped by a combination of genetic inheritance, environmental conditions and their dynamic interplay. However, as noted above, the role of genetic influences in this context remains poorly understood, and there is currently, to our knowledge, no empirical evidence directly demonstrating the extent to which gene–environment interplay shapes personalised ecologies. Insights from other domains of behavioural and psychological research nonetheless suggest that such interplay may be relevant. A substantial body of work shows that gene–environmental interplay contributes to individual differences across a wide range of traits (Hambrick and Tucker-Drob, 2015; Schellenberg, 2015; Ullén et al., 2016; Bulbena-Cabre et al., 2018; Hopwood et al., 2022; Herrera-Luis et al., 2024). Although the magnitude of these effects varies across contexts and is often smaller than the independent effects of genes or environments (Jaffee and Price, 2007; Pasman et al., 2019; Wahbeh and Avramopoulos, 2021; Virolainen et al., 2023), this evidence indicates that gene–environment interplay can shape how genetic predispositions are expressed. Extending this framework to personalised ecologies, therefore, provides a useful basis for understanding the drivers of inter-individual variation in nature experiences.

## How extinction of experience is entrenched within societies

The conceptual framework outlined above provides a basis for understanding how the extinction of experience can become entrenched within societies. Whilst a range of intergenerational trajectories are possible, here we focus on those consistent with the ongoing, global decline in nature experience. Specifically, we propose two main pathways: (1) when impoverished personalised ecologies are inherited between generations, and (2) when rich personalised ecologies fail to be transmitted between generations.

### Pathway 1: Transmission of impoverished personalised ecologies

When parents already possess limited personalised ecologies, these can often be passed on to their children. Parents with little prior experience of nature are more likely to select or remain in environments that restrict their children’s opportunities, motivations and capabilities for direct engagement with nature. For example, they may live in neighbourhoods with limited access to greenspace, choose less biodiverse residential areas (Hosaka et al., 2017) or manage domestic gardens in ways that provide little support for wildlife (Garfinkel et al., 2024). Such parents may be more likely to report greater avoidance of, or discomfort in, natural settings (Sugiyama et al., 2021), and to place lower value on interacting with nearby nature (Soga et al., 2016). This, in turn, can increase their likelihood of affiliating with social groups where engagement with nature is not actively encouraged. These environmental contexts and the personalised ecologies they foster reinforce one another, progressively compounding disconnection from nature. As a result, children raised in such contexts can develop impoverished personalised ecologies themselves.

### Pathway 2: Breakdown in the transmission of rich personalised ecologies

In other cases, parents may themselves have rich personalised ecologies, yet these are not successfully transmitted to their children. Such breakdowns can occur for two interrelated reasons. First, broader environmental changes may reduce children’s opportunities, motivations and capabilities to engage with nature compared to those available to their parents (a phenomenon widely commented on in natural history writing). For example, parents who grew up surrounded by rich biodiversity may raise their children in far more nature-depleted environments if rapid habitat loss or biodiversity decline occurs, or if families move to highly urbanised settings. In such cases, children do not inherit their parents’ experienced environments but instead adapt to the newly impoverished contexts in which they are embedded. Indeed, in recent decades, accelerating biodiversity loss, increasing urban population concentration and the spread of digital entertainment have dramatically reshaped the conditions under which childhood nature experiences occur, making it increasingly difficult for nature-rich environments to be passed on across generations.

Second, even when parents have strong orientations towards nature (which may partly reflect genetically influenced traits), these may not be realised in their children if environmental or social conditions constrain the processes through which such tendencies are expressed. This can occur through multiple, interrelated mechanisms. For example, parents who value nature may nevertheless be unable to provide nature-rich experiences if they live in neighbourhoods lacking accessible green space. Children who are inclined to seek out nature may be constrained by social norms that portray natural environments as unsafe or undesirable. Likewise, even when children show enthusiasm for plants, animals or outdoor activities, parents or teachers may lack the resources, knowledge or confidence to support these interests. More broadly, the expression of individual tendencies towards nature engagement may depend on environmental context; dispositions that might lead to frequent nature experiences in supportive settings may remain unrealised in highly constrained or degraded environments. In such situations, the potential for rich personalised ecologies is not fully expressed and may gradually erode across generations.

In either case, the intergenerational transmission of impoverished personalised ecologies may have broader societal consequences. As reduced levels of nature interaction are passed across generations, these may increasingly be perceived as normal, weakening expectations of and demand for direct engagement with nature. Whilst the extent to which such ‘shifting baselines’ operate in personalised ecologies remains unclear (Soga and Gaston, 2018), this gradual social normalisation may further reinforce the persistence of disconnection from nature over time.

## Breaking the cycle of disconnection

Importantly, our framework is not deterministic. Although intergenerational processes can contribute to the persistence of the extinction of experience, they do not imply that such trajectories are fixed or inevitable. Indeed, the same mechanisms that transmit impoverished personalised ecologies can, under more favourable conditions, support the maintenance and even enhancement of richer forms of human–nature interaction. Achieving this is likely to involve two complementary pathways: preventing the inheritance of impoverished personalised ecologies, whilst enabling the continuity and transmission of richer ones across generations.

### Promoting the transmission of rich personalised ecologies

Parents who already have rich personalised ecologies may be motivated and able – through knowledge (e.g., knowing where particular plant and animal species can be found), skills (e.g., using field guides and visual aids [e.g., binoculars] to identify wildlife) and access to resources (e.g., membership of natural history clubs) – to support their children’s experiences of nature. Yet these motivations and capabilities cannot be fully realised without environments that make such interactions possible. This highlights the importance of creating both physical and social settings that actively facilitate nature engagement. Examples include the expansion and restoration of local natural areas, especially those rich in common species, in highly urbanised regions, as well as the organisation of community-based events such as guided nature walks or biodiversity observation programmes.

### Preventing the inheritance of impoverished personalised ecologies

In practice, in many urbanised and developed societies, people with rich personalised ecologies are rare and the majority have very limited ones (Cox et al., 2017; Richards et al., 2020; Soga and Gaston, 2025). Thus, one might argue that preventing the intergenerational transmission of impoverished personalised ecologies is likely to be more effective than promoting the transmission of richer ones.

Such prevention cannot be achieved solely by expanding greenspace, which is currently a major focus of policy (e.g., World Health Organization, 2017; CityHealth, 2023). Children with impoverished personalised ecologies often grow up in environmental and social conditions inherited from preceding generations that limit exposure to biodiversity, reduce opportunities for direct nature experience and place less emphasis on nature in everyday life. Within these contexts, children may develop substantially lower motivation and capability to engage with nature, reducing the effectiveness of interventions that rely primarily on increasing opportunity. Indeed, affective and cognitive factors, such as individuals’ desire to engage with nature, can in some cases be more influential than opportunity alone in shaping nature engagement (e.g., Lin et al., 2014; Bloemsma et al., 2018; Soga and Akasaka, 2019).

Accordingly, policies should recognise that children growing up in nature-deprived contexts may require support that goes beyond increasing opportunity alone, particularly through strengthening motivation and capability. A key entry point is parents, who play a central role in shaping children’s behaviours, attitudes and everyday environments. This can be achieved through two complementary approaches: reducing barriers to engagement and enhancing the attractiveness of nature experiences. Reducing barriers perceived by parents (particularly in urban contexts) can facilitate engagement, for example, by addressing safety concerns through supervised or well-designed natural spaces. Constraints related to distance can also be mitigated by integrating attractive and biodiverse elements into everyday environments, such as street greenery or school grounds where children spend much of their time. Equally important, strengthening parental motivation can further promote engagement with nature, for instance by framing nature experience as a health-promoting behaviour supported by growing evidence (e.g., nature-based interventions; Shanahan et al., 2019).

Beyond parents, children’s environments are also shaped by a wider range of social actors. Multiple actors can therefore contribute to enhancing capability, opportunity and motivation in ways that may compensate for limitations within the family environment. Conservation organisations, museums and botanical gardens can provide accessible and well-supported opportunities for engagement with nature (Stokes, 2006). Community-based programmes (such as intergenerational outdoor activities, nature clubs and citizen science projects, including those linked to school activities) can build knowledge and skills (D’Amore, 2016). Schools can further play a critical role by providing structured nature experiences and fostering motivation in ways that extend beyond the classroom and into family life; such influences may also shape parental attitudes and behaviours (see Boudet et al., 2016 for evidence of child-to-parent transmission in pro-environmental behaviours). As such, the transmission of personalised ecologies between parents and children may be bidirectional rather than unidirectional.

Collectively, these initiatives may extend beyond individuals and families, with effects that scale up to broader societal contexts and contribute to the intergenerational transmission of richer personalised ecologies. When implemented consistently and at scale, such efforts may also help to foster relational values, defined as the emotional, cultural and ethical dimensions of people’s relationships with nature (Chan et al., 2018). The accumulation of these values at the societal level can, in turn, strengthen shared norms and practices that support nature engagement, thereby contributing to the intergenerational continuity of enabling environments for personalised ecologies.

Achieving such outcomes, however, may require intervention strategies that extend beyond conventional access- or knowledge-based approaches. Whereas conventional interventions often seek to increase the frequency of nature contact or improve knowledge about biodiversity, relational-value-oriented interventions may place greater emphasis on cultivating meaningful and identity-relevant relationships with nature (see Kleespies and Dierkes, 2020). Such interventions may involve structured and reflective forms of engagement, including stewardship-based activities, cultural practices or place-based learning, that encourage participants to perceive nature as part of their identity, local community and sense of responsibility towards the environment (Uehara et al., 2020; Tajima et al., 2026).

To advance these efforts, it is essential to evaluate interventions as they are implemented. At present, the large-scale and long-term effects of such approaches remain poorly understood. Iterative evaluation and refinement of interventions will therefore be critical. Future research should also examine heterogeneity in responses to interventions, including how differences in personal traits shape their outcomes. Whilst some individuals may have a lower baseline orientation towards nature, potentially influenced by genetic factors, this should not be interpreted as implying fixed limits on engagement or immutable differences in responsiveness. Even where such predispositions exist, their influence is likely to depend heavily on the social and environmental settings in which individuals develop and live. Importantly, the primary leverage for policy and practice lies in modifying the environmental, institutional and social conditions that structure opportunities and motivations for nature engagement. Supportive contexts can therefore broaden participation across diverse groups, including those who may initially be less inclined to engage with nature.

## Implications

The perspectives developed here have important implications for both research and practice. From a research perspective, our framework highlights the need to incorporate intergenerational dynamics into studies of personalised ecologies. As suggested in this article, and partly supported by empirical evidence, individuals’ nature experiences are likely to be shaped, at least in part, by those of preceding generations. Ignoring such intergenerational influences may therefore lead to misinterpretation of both the current state of personalised ecologies and the factors that drive them. Although incorporating intergenerational processes presents practical challenges, it also opens up new avenues for developing and testing novel hypotheses, reinterpreting existing findings and generating more robust predictions about the long-term dynamics of personalised ecologies (see the list of key questions in Table 1).

**Table 1.** Examples of key research questions for understanding the intergenerational transmission of the extinction of experience Table 1. long description.

Theme	Key questions
Mechanisms of intergenerational transmission	What are the relative contributions of genetic, environmental, gene–environment correlation (rGE) and gene–environment interaction (G × E) processes in shaping the intergenerational transmission of personalised ecologies? Under what conditions can parents with impoverished personalised ecologies still foster richer personalised ecologies in their children? How do cultural contexts shape the ways personalised ecologies are transmitted across generations? To what extent can children’s own nature experiences influence or enrich the personalised ecologies of their parents (child-to-parent transmission)?
Strategies and evaluation of interventions to prevent the extinction of experience	What kinds of messages, incentives or interventions are most effective in motivating parents with limited personalised ecologies to support their children’s engagement with nature? Can schools or teachers compensate for the limited personalised ecologies of parents, and if so, how is this most effective? How long does it take for interventions designed to enrich personalised ecologies to produce measurable effects across generations?

Addressing these questions will require broader interdisciplinary collaboration across conservation biology, behavioural genetics, psychology and education, alongside the application of diverse methodological approaches. These include longitudinal and multi-generational studies that track changes in personalised ecologies over time, family-based or cross-cohort approaches that enable comparisons across generations and intervention or quasi-experimental designs that assess how changes in environmental conditions influence opportunities, motivation and capability for nature engagement (e.g., school-based programmes, community interventions or natural experiments arising from changes in urban green space provision). In addition, approaches from behavioural genetics, such as twin or pedigree designs, can help to disentangle genetic and environmental influences on variation in personalised ecologies.

Our framework also has important implications for policy and practice. Interventions aimed at preventing the intergenerational transmission of impoverished personalised ecologies may contribute not only to sustaining human–nature interactions at the societal level but also to reducing social inequalities in nature experience. Households with limited access to safe, biodiverse and culturally relevant natural environments (often associated with lower socioeconomic status; Leong et al., 2018) may be more likely to transmit impoverished personalised ecologies across generations. As a result, disadvantages may accumulate over time, with consequences for the health and well-being of affected individuals and communities. In this context, it may be important to consider how interventions can better reach and support those who are more likely to experience such cycles. Importantly, preventing such cycles is not only relevant for addressing visible inequalities within the current generation, but also for avoiding less visible forms of intergenerational injustice, whereby future generations are effectively constrained to inherit impoverished personalised ecologies relative to those experienced today (McKinnon and Gardiner, 2026).

Sustained implementation of these efforts has the potential to break self-reinforcing cycles of disconnection and instead initiate positive feedback across generations, in which enriched personalised ecologies are maintained and transmitted rather than eroded. Children who grow up with richer personalised ecologies are more likely to carry these experiences into adulthood, shaping their choices and behaviours as parents, educators and community members (Gaston et al., 2023; Soga & Gaston, in press). In turn, they contribute to creating environments that support opportunities, motivation and capability for nature engagement in subsequent generations, thereby sustaining and strengthening personalised ecologies over time.

## Concluding remarks

Evidence shows that people’s personalised ecologies are diminishing across much of the world (Soga and Gaston, 2023a), raising concerns for both human health and conservation. This concern has grown at a time when the world faces an unprecedented biodiversity crisis. Undoubtedly, species extinctions and the extinction of experience are tightly linked: species loss reduces opportunities for personal experiences of nature, whilst the decline of personalised ecologies can, in turn, accelerate species declines. One might therefore argue that preventing the extinction of experience is as essential to global biodiversity conservation as preventing species loss itself. Indeed, biodiversity conservation ultimately depends on shaping people’s decisions and behaviour (Veríssimo et al., 2024), and personalised ecologies can play a crucial role in this process (Gaston et al., 2023; Soga & Gaston, in revision). Breaking the intergenerational cycle of extinction of experience and transforming it into a reinforcing virtuous cycle of human–nature interactions will be vital for the future of both biodiversity and humans.

## Data Availability

Data availability is not applicable to this article as no new data were created or analysed in this study.

## Table 1. Long description

The table has two columns: Theme and Key questions. The first row theme is Mechanisms of intergenerational transmission. Its key questions are: What are the relative contributions of genetic, environmental, gene–environment correlation (r G E) and gene–environment interaction (G times E) processes in shaping the intergenerational transmission of personalised ecologies; Under what conditions can parents with impoverished personalised ecologies still foster richer personalised ecologies in their children; How do cultural contexts shape the ways personalised ecologies are transmitted across generations; To what extent can children’s own nature experiences influence or enrich the personalised ecologies of their parents, described as child-to-parent transmission. The second row theme is Strategies and evaluation of interventions to prevent the extinction of experience. Its key questions are: What kinds of messages, incentives or interventions are most effective in motivating parents with limited personalised ecologies to support their children’s engagement with nature; Can schools or teachers compensate for the limited personalised ecologies of parents, and if so, how is this most effective; How long does it take for interventions designed to enrich personalised ecologies to produce measurable effects across generations.

Navigate back to Table 1..
