# Supplementary material for: Breaking the intergenerational cycle of extinction of experience: Actions needed and pathways forward
Source: Camb Prism Extinct. 2026 May 26;4:e7. doi: 10.1017/ext.2026.10015 (PMC13312371; doi:10.1017/ext.2026.10015)
Supplement: Peer review history

# Reviewer report: Review: Breaking the intergenerational cycle of extinction of experience: Actions needed and pathways forward — R0/PR2

**DOI:** 10.1017/ext.2026.10015.pr2

This Perspective paper continues a series of important perspectives and studies by Soga and Gaston on the “extinction of experience”, the progressive decline in direct, sensory interactions between people and nature. Building on their earlier conceptual and empirical contributions, the paper extends the discussion to an explicitly intergenerational dimension by proposing a behavioural-genetic framework for understanding how “personalised ecologies” may be inherited through genetic, environmental, and interactive pathways. The authors argue that such processes can generate self-reinforcing cycles of disconnection from nature and call for interventions that address both the inheritance of impoverished experiences and the breakdown of rich ones.

While I greatly appreciate the authors’ long-standing contribution to advancing this field, I am concerned that this paper, rather than moving it forward, builds on a limited and only partly established empirical foundation. The integration of behavioural genetics into the extinction of experience framework remains highly speculative, relying on assumptions that are not yet sufficiently supported by evidence. In my view, the paper should be substantially revised to frame this as a suggestive and exploratory idea rather than a well-established framework. Presenting it in this way would more accurately reflect the current state of knowledge, highlight existing uncertainties, and invite further investigation into a concept that, while interesting, remains largely hypothetical.

Comments

The paper builds directly on the authors’ well-known body of work on the extinction of experience and personalised ecologies. While this continuity is valuable, the conceptual advance offered here is limited. The main addition is the integration of behavioural genetics into the framework, which in my view (see comment below), remains more metaphorical than operational. The proposed genetic and gene–environment correlation (rGE) mechanisms are described largely in speculative terms, with limited empirical evidence that such inheritance processes shape human–nature interactions. The paper would benefit from a clearer explanation of how these mechanisms could realistically be tested and differentiated from purely social or cultural transmission pathways.

My main concern regarding this perspective is that the proposed genetic pathway, which forms the central innovation of the paper, remains conceptually weak and empirically unsupported. While it is plausible that fear-related responses to natural stimuli (biophobia) could have been shaped by evolutionary pressures, there is still no direct genetic evidence identifying heritable components or mechanisms underlying such traits. The theoretical rationale for selection is more defensible here, as avoidance of dangerous animals could have conferred survival advantages, yet even this remains unproven at the genetic level and poorly studied with very few studies exploring this hypothesis. For biophilia, the case is far weaker: the hypothesis of an innate attraction to nature lacks both mechanistic and evolutionary justification, and as Joye and De Block (2011) argue, it remains conceptually ambiguous and empirically unsubstantiated. They further criticise the biophilia hypothesis for relying on speculative evolutionary narratives, circular reasoning, and a tendency to reinterpret cultural or learned preferences as innate biological dispositions. By borrowing the vocabulary of genetics without empirical support, the framework risks introducing unnecessary complexity and potentially misleading readers unfamiliar with behavioural genetics. In my view, it is particularly important that a perspective paper does not introduce such conceptual biases, as this could lead future research in the wrong direction and distort the field’s theoretical development. To avoid this and to make the perspective valuable and credible, it is essential to establish a clear rationale for the proposed genetic framing, explaining the biological mechanisms through which such inheritance could plausibly operate, highlight limitations and knowledge gaps. This can help engage the perspective in a much deeper and more critical discussion of this pathway, even in the absence of direct empirical evidence. Yet, I also wonder whether the existing evidence is sufficient to justify building an entire perspective around this genetic argument.

Joye, Y. and De Block, A., 2011. ‘Nature and I are two’: A critical examination of the biophilia hypothesis. Environmental Values, 20(2), pp.189-215.

Large parts of the argument reiterate ideas already published by the same authors in various platforms. The novelty here appears to lie mainly in the genetic framing, yet this section remains underdeveloped and not well backed up with empirical evidence from genetics (as I previously highlighted). The journal might wish to consider whether the contribution is sufficiently distinct to justify a standalone Perspective.

The essay focuses heavily on genetic and environmental pathways but pays little attention to broader socio-cultural or structural processes known to drive the extinction of experience, such as urban design, inequality in access to green space, technological shifts, and environmental education systems. These factors are acknowledged only briefly, yet they likely explain much more of the intergenerational variation than genetics. The argument would benefit from situating the framework within a richer socio-ecological context and clarifying its added explanatory value relative to established social theories.

In lines 103-107, the authors explicitly state that the paper focuses only on positive or neutral human–nature interactions, excluding negative experiences. I find this restriction difficult to justify, as omitting the negative dimension risks presenting an incomplete and somewhat idealised view of the extinction of experience. To some extent, the perspective already acknowledge that both biophilia and biophobia can be intergenerationally transmitted, yet this dimension remains underdeveloped. If the proposed genetic and behavioural framework is valid, it should apply equally to positive and negative pathways of inheritance. Therefore, I would strongly encourage the authors to integrate both aspects into the conceptual model rather than keep them separate. Doing so would make this perspective more balanced, comprehensive, and theoretically coherent, avoiding the impression of a one-sided or selective treatment of the topic.

The authors criticise existing initiatives for being “short-term interventions” (line 92), yet the paper does not clearly demonstrate what novel or long-term strategies are proposed instead. The suggested actions later in the text, such as expanding green spaces, educational programmes, and community activities, are largely similar to those already discussed in the literature and do not convincingly differ from the short-term approaches they criticise. Moreover, it remains unclear to what extent the effectiveness of such interventions in mitigating the extinction of experience has been empirically established (both short and long term). The authors should either ground their claims with supporting empirical evidence or call for more research to address this gap. In addition, they should clarify what genuinely new or distinctive interventions they advocate, and justify why these are expected to generate more durable, intergenerational effects than existing measures.

Additional major concern stem from the pathways the perspective suggests. The conceptual framework distinguishes two pathways: the inheritance of impoverished personalised ecologies and the failure to transmit rich ones. However, this formulation seems incomplete and overly one-sided. It implicitly assumes that negative cycles of disconnection are the dominant or default pattern, while overlooking the possibility that positive cycles—where rich personalised ecologies are successfully transmitted, or where impoverished ones are disrupted and reversed—may also occur. In my view, a truly comprehensive framework should account for all four potential pathways and explore the mechanisms underlying each. For instance, why would impoverished ecologies be more easily transmitted than rich ones? What evidence supports the claim that limited personalised ecologies are often passed to children, as stated around line 188? Similarly, if rich experiences sometimes fail to be transmitted, what explains this asymmetry? Without addressing these questions, the model risks appearing deterministic, incomplete, and potentially misleading. Moreover, by focusing almost exclusively on the negative cycle, the framework overlooks the positive forces and counteracting processes that might sustain or restore connection to nature across generations. This section would benefit from a more balanced and mechanistically grounded discussion that also considers positive intergenerational trajectories of nature experience.

I find the sections on breaking the cycle of disconnection from nature not to be particularly useful, as they largely reiterate ideas already presented in previous publications. The subsection on promoting the transmission of rich personalised ecologies does not introduce genuinely new ideas beyond what has been discussed in earlier literature by the authors and others. The recommendations remain broad and repeat familiar actions to enhance nature experiences without clearly demonstrating how these could foster long-term, intergenerational transmission of nature connection. In its current form, the discussion lacks conceptual and practical novelty and would benefit from a clearer articulation of what is new and how these strategies differ from previous work. The subsection on preventing the intergenerational inheritance of impoverished personalised ecologies similarly emphasises expanding access to green spaces, promoting environmental education, and strengthening institutions such as museums and botanical gardens. In my view what can be useful here is a discussion on relational values, the emotional, cultural, and ethical dimensions of people’s connections with nature. I do not see this aspect developed or even acknowledged, yet it could offer a much stronger foundation for understanding how lasting relationships with nature are formed. Developing an approach that integrates and operationalises relational values within interventions aimed at preventing the intergenerational transmission of disconnection from nature would mark a significant shift beyond access- or knowledge-based strategies, toward fostering deeper and more enduring human–nature relationships that can be sustained across generations.

In the implications the authors state that “much of the existing literature has focused on proximate, within-generation processes, while these short-term dynamics are in fact embedded within longer, self-reinforcing cycles that unfold across generations”. Yet, if these processes are already embedded within one another, it remains unclear what the conceptual or empirical novelty of this perspective is. The paper does not convincingly demonstrate how recognising these broader temporal dimensions provides genuinely new insight or opens up distinct avenues for research. To strengthen this argument, the authors should clarify what specific questions or hypotheses emerge from adopting this intergenerational framing and how each proposed avenue of research could advance the field beyond existing work.

Many of the references are drawn from the authors’ own extensive body of work on the extinction of experience, several of which rely more on conceptual or circumstantial evidence than on empirical data. While I fully recognise the authors’ substantial and lasting contribution to this field, the paper would benefit from broader engagement with independent, empirically grounded studies. This should include literature on environmental socialisation, childhood development, and intergenerational learning, as well as experimental and intervention-based research assessing what effectively enhances nature experiences and interactions. A stronger integration of such evidence would make the argument more robust, balanced, and practically informative.

The impact statement partly duplicates the abstract and might be shortened or reframed to focus on the unique contribution of the paper.

Table 1 is useful but descriptive; it could be more valuable if linked to specific methodological approaches, interventions or metrics.

Figure 1 is visually clear but conceptually simplistic. It repeats the tripartite structure (genetic, environmental, and rGE pathways) without illustrating how these interact dynamically across time or scales. The figure could be improved by showing feedback loops and intervention points (e.g., how education or urban design can modify rGE effects). As presented, it adds little beyond the textual description.

# Reviewer report: Review: Breaking the intergenerational cycle of extinction of experience: Actions needed and pathways forward — R0/PR3

**DOI:** 10.1017/ext.2026.10015.pr3

**Authors:** Barry Brook

**Affiliations:** School of Natural Sciences, University of Tasmania, Australia

This is a clear, compact Perspective that sits well in the human factors angle of the journal. The MS argues that the extinction of experience (declining direct, everyday interactions with nature) is self-reinforcing across generations, and it proposes a conceptual framework for intergenerational transmission of “personalised ecologies” grounded in behavioural genetics (genetic pathways, environmental pathways, and gene–environment correlation). It then distinguishes two reinforcing pathways by which extinction of experience becomes entrenched (transmission of impoverished personalised ecologies; and breakdown in transmission of rich personalised ecologies) and outlines broad strategies to interrupt the cycle. The framing is timely, the writing is generally accessible, and the two display items (Table 1 and Figure 1) are clear, the figure is attractive, and they’re clearly relevant to the core message.

At the same time, I think the piece currently has two categories of substantive issues. Major: conceptual balance and positioning (especially around what behavioural genetics adds, and how to avoid deterministic readings). Minor: problems in the reference list (missing references, duplicated-year citation handling, and one visibly corrupted entry). These need addressing before publication.

Conceptual contribution and balance

The behavioural genetics lens is an interesting choice, but the manuscript needs to work harder to persuade a broad extinction/conservation readership that this lens adds explanatory traction beyond standard accounts of intergenerational cultural transmission, social learning, and structural constraints (e.g., urban form, safety norms, socioeconomic inequality, schooling). Right now, the “environmental pathways” section reads like a conventional (and convincing) explanation of intergenerational similarity, whereas the “genetic pathways” and rGE sections are more speculative and risk being interpreted as implying a stronger evidence base than exists for this specific phenomenon. The authors do acknowledge that genetic contributions/rGE are “poorly understood” and call for disentangling them, but the way the text is written still leans toward presenting plausible mechanisms without a clear boundary between known in behavioural genetics generally and demonstrated for personalised ecologies specifically.

Relatedly, because extinction of experience is a concept often used in policy/education contexts, I think it is important to explicitly manage the ethical and interpretive risks of invoking genetic transmission. Even if scientifically defensible, a behavioural genetics framing can be misread as (a) implying immutability (“it’s genetic, therefore hard to change”) or (b) shifting responsibility from institutions and environments onto families/individuals. The manuscript would benefit from an explicit paragraph (likely in the “Conceptual framework” section or at the start of “Implications”) stating that heritability does not imply inevitability, that heritability is population- and context-dependent, and that policy leverage points still primarily operate through modifiable environments and institutions. As written, phrases such as “genetic predisposition to enjoy nature” (around the passive rGE example) could be softened or contextualised to avoid deterministic interpretations.

There is also a conceptual omission that will likely irk behavioural genetics readers: the MS focuses on rGE but does not discuss gene × environment interaction (GxE). If the aim is to import core behavioural genetics ideas, it’s at least worth acknowledging that genetic propensities may be expressed differently across environmental contexts (which is directly relevant to the authors’ emphasis on opportunity/motivation/capability constraints). Even a short note distinguishing rGE (selection into environments correlated with genotype) from GxE (environment moderates genetic effects) would improve accuracy and reduce the risk of presenting rGE as the sole “interplay” mechanism.

Scope, participants, and intergenerational framing

The argument is framed largely around parent–child transmission (Figure 1 is explicitly Parent and Child), but much intergenerational continuity/discontinuity in nature experience is mediated by broader kin networks and institutions (grandparents, extended family; schools and peer groups; youth organisations; religious/cultural practices; community safety norms). The text does mention social contexts and schools, but the intergenerational model would be stronger if it explicitly recognised that inheritance of personalised ecologies can occur through multiple social pathways beyond nuclear-family parenting. This seems important because it has direct implications for interventions: if non-parental pathways are important, then breaking the cycle can be accomplished even when parents have impoverished personalised ecologies, via school-based and community-based scaffolding.

The two-pathway structure (transmission of impoverished personalised ecologies vs failure to transmit rich ones) is quite intuitive and helpful, but the Perspective could be clearer about how these pathways relate to the behavioural genetics framing. In places, the discussion shifts from the genetic/environment/rGE taxonomy to broader societal changes (urbanisation, digital entertainment) without clearly mapping these drivers onto opportunity/motivation/capability or onto the transmission mechanisms. A small tightening, e.g., explicitly linking each pathway to which components of opportunity/motivation/capability are being constrained and by whom. This would make the framework feel less like two parallel narratives.

Evidence base and overstatement risk

A Perspective can be conceptual, but it still needs to be careful about the strength of claims. A few spots read as stronger than the cited evidence likely supports, for example where the text implies that individuals with biophobic tendencies might “modify their surroundings in ways that are harmful to nature” via pesticides/clearing (Conceptual framework --> Genetic pathways). This may be plausible, but it would be stronger if the text distinguished between hypothesised causal pathways and demonstrated behavioural outcomes, or if it briefly noted that these links may be mediated by socioeconomic and cultural factors (e.g., gardening practices, norms, pest management). Similarly, biophilia is presented as innate (citing Wilson/Kellert), but that hypothesis remains debated (!); if the paper is using it as shorthand for trait-like nature orientation, it may be safer to use more neutral wording (e.g., “individual differences in affinity for nature”) and position biophilia as one interpretation rather than a settled fact.

Interventions section: specificity and criticality

The “Breaking the cycle” section is sensible but somewhat generic at present (expand/restore local natural areas; community events; museums/botanical gardens; nature-based public health framing; intergenerational clubs/citizen science). For a Perspective in this journal, I think that’s acceptable, but it would be stronger if the authors added at least one paragraph that critically evaluates why common interventions have limited long-term intergenerational effects, and what design principles would make interventions more likely to persist across generations. Table 1 provides research questions; something analogous in the narrative for intervention design (even without adding a new table/figure) would sharpen the “actions needed and pathways forward” part of the title.

Minor points

Table 1 is genuinely useful as a future research prompt. That said, the two themes are both framed as research questions, but several questions are actually evaluation/implementation science questions. That’s not exactly a problem, but the authors might consider whether the theme labels could better reflect that (e.g., explicitly including intervention evaluation under the second theme).

Figure 1 communicates the intended message quickly; I like it. But two quibbles to consider. First, accessibility: it relies on colour (blue vs pink arrows) for the two transmission types; might be hard for colour-blind readers to distinguish? Better labels and/or line styles would help. Second, regarding conceptual precision: representing “gene–environment correlation (rGE)” as a central labelled element is fine, but I worry a bit that the figure could be interpreted as rGE being an independent “thing” transmitted to the child rather than a class of processes generating correlation. A minor edit to the caption language or diagram structure would reduce that risk.

References:

The Ceballos et al. (2015) reference is incomplete, and the journal/volume information (“Science Advances, 1:e1400253.”) is, I think, incorrectly concatenated onto the end of the CityHealth (2023) ref.

Several in-text citations are missing from the reference list, e.g., Chang et al. (2022), Samus et al. (2022), and Hoggett et al. (2024). You also cite “Gaston, 2024” but I think you mean 2023?

There are two different Soga & Gaston (2023) papers listed in the references (One Earth; Conservation Letters), but they are not disambiguated as 2023a/2023b in the reference list, and the in-text citations appear as “Soga & Gaston, 2023” without an a/b suffix.

“Samus et al. 2022” is missing a comma after “al.”.

Overall Recommendation

I see clear merit in publishing this Perspective, but I would recommend a major revision focused on strengthening the conceptual balance around behavioural genetics (clearer demarcation of what is evidenced vs hypothesised for personalised ecologies; a brief discussion of interpretive/ethical cautions; and ideally a short acknowledgement of G×E and broader social transmission pathways). Plus those minor fixes to the display items, refs etc. With those changes, the piece should make a strong conceptual contribution for this journal.

# Editor report: Recommendation: Breaking the intergenerational cycle of extinction of experience: Actions needed and pathways forward — R0/PR4

**DOI:** 10.1017/ext.2026.10015.pr4

**Affiliations:** Durrell Institute of Conservation and Ecology, University of Kent, United Kingdom of Great Britain and Northern Ireland

Firstly, I would like to again sincerely apologise for the time it has taken to complete the review of this manuscript. I appreciate your patience during this process and regret any inconvenience the delay may have caused.

Having considered the manuscript, myself and the responses of the two reviewers I believe the manuscript is suitable for publication following Major Corrections, and hope the feedback provided is helpful in moving the manuscript forward.

Both reviewers agree the Perspective is clearly written, timely, and offers a useful intergenerational framing of the “extinction of experience,” with effective visuals and a potentially valuable conceptual structure. However, both raise major concerns about the central behavioural genetics framing, arguing it is speculative, insufficiently evidenced, and risks deterministic or potentially misleading interpretations. They recommend clarifying what is empirically supported versus hypothetical, better integrating broader socio-cultural drivers, and strengthening conceptual balance (including gene–environment interaction and non-family pathways).

# Editor report: Decision: Breaking the intergenerational cycle of extinction of experience: Actions needed and pathways forward — R0/PR5

**DOI:** 10.1017/ext.2026.10015.pr5

**Affiliations:** Durrell Institute of Conservation and Ecology, University of Kent, United Kingdom of Great Britain and Northern Ireland

No accompanying comment.

# Reviewer report: Review: Breaking the intergenerational cycle of extinction of experience: Actions needed and pathways forward — R1/PR7

**DOI:** 10.1017/ext.2026.10015.pr7

**Authors:** Barry Brook

**Affiliations:** School of Natural Sciences, University of Tasmania, Australia

Overall, I’m happy with the revision. The framework is more "hypothesis‑generating: and the behavioural genetics material (and explicit incorporation of GxE interaction) is better bounded.

There are still a few lingering issues:

-- On the ethical/interpretive risk of invoking genetics (immutability, blame‑shifting), the revision has partially addressed my concern, but there’s still a bit of mismatch between the revised MS and what’s stated in the response letter. The point that heritability is population/context dependent, and policy leverage sits mainly with environments/institutions, is still only partly present in the MS text. Given that “extinction of experience” is often used in public‑facing and policy settings, I still think a short, explicit paragraph (or a couple of sentences) that makes these points in unambiguous language is desirable.

-- The relational values material has been added, but it remains very brief (really just a nod rather than an integrated pillar of the intervention logic!). It needs more of a conceptual expansion rather than a tokenistic hat tip. Maybe one more paragraph that explains how relational‑values interventions would be designed/evaluated differently from access/knowledge interventions?

-- in the Gene‑environment interplay section you state there is “no empirical evidence directly demonstrating” gene-environment interplay for personalised ecologies and then add in brackets “(equally, there do not appear to be published studies that have sought such effects but failed to find them).” That second part reads as quite defensive and isn’t really informative. I’d recommend deleting it or replacing it with a cleaner statement about evidence gaps and the need for targeted study designs (which you already discuss later).

-- I still saw some quirks with the refs, e.g. Palviainen et al., 2025 not in ref list? Soga et al 2018 and Yamanoi et al 2021 seem to be the same papers (same title and journal but different author order and years: all very confusing!). Soga & Gaston 2022 needs a 2022a and 2022b, also for Chang et al 2022.

# Editor report: Recommendation: Breaking the intergenerational cycle of extinction of experience: Actions needed and pathways forward — R1/PR8

**DOI:** 10.1017/ext.2026.10015.pr8

**Affiliations:** Durrell Institute of Conservation and Ecology, University of Kent, United Kingdom of Great Britain and Northern Ireland

Thank you for submitting corrections to your previous manuscript. The revised manuscript has gone a long way to addressing a number of issues raised. A previous review who has now reviewed your revised manuscript has raised a few minor issues that remain and I agree with. I am confident you will be able to easily address before I can accept. I look forward to seeing the revised manuscript.

# Editor report: Decision: Breaking the intergenerational cycle of extinction of experience: Actions needed and pathways forward — R1/PR9

**DOI:** 10.1017/ext.2026.10015.pr9

**Affiliations:** Durrell Institute of Conservation and Ecology, University of Kent, United Kingdom of Great Britain and Northern Ireland

No accompanying comment.

# Editor report: Recommendation: Breaking the intergenerational cycle of extinction of experience: Actions needed and pathways forward — R2/PR11

**DOI:** 10.1017/ext.2026.10015.pr11

**Affiliations:** Durrell Institute of Conservation and Ecology, University of Kent, United Kingdom of Great Britain and Northern Ireland

I am satisfied with the corrections made and agree with the handling editor that it can now be accept. I look forward to seeing it in print.

# Editor report: Decision: Breaking the intergenerational cycle of extinction of experience: Actions needed and pathways forward — R2/PR12

**DOI:** 10.1017/ext.2026.10015.pr12

**Affiliations:** Durrell Institute of Conservation and Ecology, University of Kent, United Kingdom of Great Britain and Northern Ireland

No accompanying comment.
